# Switching From Aflibercept 2 mg to 8 mg in Vitrectomized Eyes With Neovascular Age-Related Macular Degeneration

**DOI:** 10.7759/cureus.93645

**Published:** 2025-10-01

**Authors:** Ryuto Tamai, Tomohiro Nizawa, Yuto Kawamata, Takehito Iwase, Takayuki Baba

**Affiliations:** 1 Department of Ophthalmology and Visual Science, Chiba University Graduate School of Medicine, Chiba, JPN; 2 Department of Ophthalmology, Chiba Rosai Hospital, Chiba, JPN

**Keywords:** aflibercept 8 mg, intravitreal pharmacokinetics, neovascular age-related macular degeneration, treat-and-extend, vitrectomy

## Abstract

Vitrectomy may alter intravitreal pharmacokinetics through the removal of the vitreous gel, potentially accelerating the clearance of anti-vascular endothelial growth factor (VEGF) agents. Clinical trials and most real-world studies on neovascular age-related macular degeneration (nAMD) generally exclude vitrectomized eyes, and the efficacy of anti-VEGF therapy in this subgroup remains unclear. Aflibercept 8 mg, approved in Japan in 2024, delivers four times the dose of the conventional 2 mg formulation and is designed to improve durability and extend treatment intervals. We report three vitrectomized eyes from three patients with nAMD who exhibited persistent or recurrent exudation despite short-interval (≤8 weeks) aflibercept 2 mg therapy under a treat-and-extend regimen. Switching to aflibercept 8 mg led to the resolution of fluid in all cases and enabled interval extension. No ocular or systemic adverse events were observed. These findings suggest that aflibercept 8 mg can achieve improved anatomical outcomes and greater treatment durability in vitrectomized eyes with nAMD, thereby potentially reducing the treatment burden in this challenging subgroup. Larger prospective studies are required to validate these findings.

## Introduction

Neovascular age-related macular degeneration (nAMD), characterized by macular neovascularization (MNV), is one of the leading causes of severe visual impairment among the elderly worldwide [1]. Intravitreal injections of anti-vascular endothelial growth factor (VEGF) agents have substantially improved both visual and anatomical outcomes, as demonstrated in large randomized clinical trials [2-7]. Currently, anti-VEGF therapy is the first-line treatment for nAMD, and the treat-and-extend regimen is widely adopted to balance efficacy with a reduced treatment burden.

However, most pivotal trials and real-world studies have excluded eyes that have undergone pars plana vitrectomy (PPV), leaving uncertainty regarding treatment efficacy in this subgroup. The vitreous gel, composed of collagen and glycosaminoglycans, acts as a barrier to drug diffusion [8], and its removal may accelerate intraocular drug clearance and shorten the half-life of anti-VEGF agents. Previous reports, including those by Mun et al. [9] and several case studies [10-13], have shown that anti-VEGF therapy can still provide anatomical and visual benefits after vitrectomy; however, the evidence remains limited.

In Japan, multiple anti-VEGF agents are available, including ranibizumab [3], aflibercept (2 mg [4] and 8 mg [5]), brolucizumab [6], and faricimab [7]. The 8 mg aflibercept formulation, approved in April 2024, delivers four times the dose of the conventional 2 mg injection and has been shown in the PULSAR trial to maintain efficacy with extended dosing intervals in treatment-naïve nAMD [5]. Its high molar concentration and prolonged VEGF suppression may offer advantages in vitrectomized eyes, where drug clearance may be increased.

Here, we report three cases of vitrectomized eyes with nAMD in which switching from aflibercept 2 mg to aflibercept 8 mg improved exudative control and enabled interval extension.

## Case presentation

We reviewed all vitrectomized eyes with nAMD that were treated at Chiba University Hospital between September and December 2024. During this period, 20 eyes from 20 patients received anti-VEGF therapy. Among these, three eyes of three patients were switched from aflibercept 2 mg to aflibercept 8 mg, and all were included in this report. Each patient completed at least six months of follow-up after the switch. The cases are presented in detail in Table 1.

**Table 1:** Baseline characteristics and outcomes before and after switching from aflibercept 2 mg to 8 mg in vitrectomized eyes with nAMD *Age at switching from aflibercept 2 mg to 8 mg BCVA, best-corrected visual acuity; IVA, intravitreal aflibercept; IVF, intravitreal faricimab; IVR, intravitreal ranibizumab; MNV, macular neovascularization; nAMD, neovascular age-related macular degeneration; PCV, polypoidal choroidal vasculopathy; PDT, photodynamic therapy; PPV, pars plana vitrectomy; IRF, intraretinal fluid; SRF, subretinal fluid

	Age*	Sex	MNV subtype	Cause of PPV	Previous treatment for nAMD before PPV	Time between PPV and first injection after PPV (weeks)	Total of treatments for nAMD before switching (times)	Time between PPV and switching (weeks)	Before switching			6 months after switching		
									BCVA	Intervals (weeks)	Exudation (Y:yes, N:no)	BCVA	Intervals (weeks)	Exudation (Y:yes, N:no)
Case 1	85	Male	Type 1 MNV	Posterior capsule rupture	IVR ×1	4	IVR ×58, IVA ×53, PDT ×1	610	20/32	5	N	20/25	8	N
Case 2	78	Male	Type 1 MNV	Lens dislocation (Zinn’s zonule rupture)	IVA ×15	6	IVA ×60	391	20/32	8	Y (SRF+)	20/40	8	Y (SRF-)
Case 3	84	Female	PCV	Epiretinal membrane	None	227	IVF ×3, IVA ×13, PDT ×1	670	20/32	6	Y	20/25	8	N

Case 1

An 85-year-old man with type 1 MNV had received anti-VEGF therapy for approximately 11 years. During the second intravitreal injection (ranibizumab), a lens injury occurred, leading to posterior capsule rupture and cataract progression. The patient subsequently underwent cataract extraction, PPV with posterior vitreous detachment (PVD) induction, and on-the-bag intraocular lens (IOL) implantation. His treatment history included 58 ranibizumab injections, followed by 53 aflibercept injections (2 mg), and one session of photodynamic therapy (Table 1).

**Figure 1:**
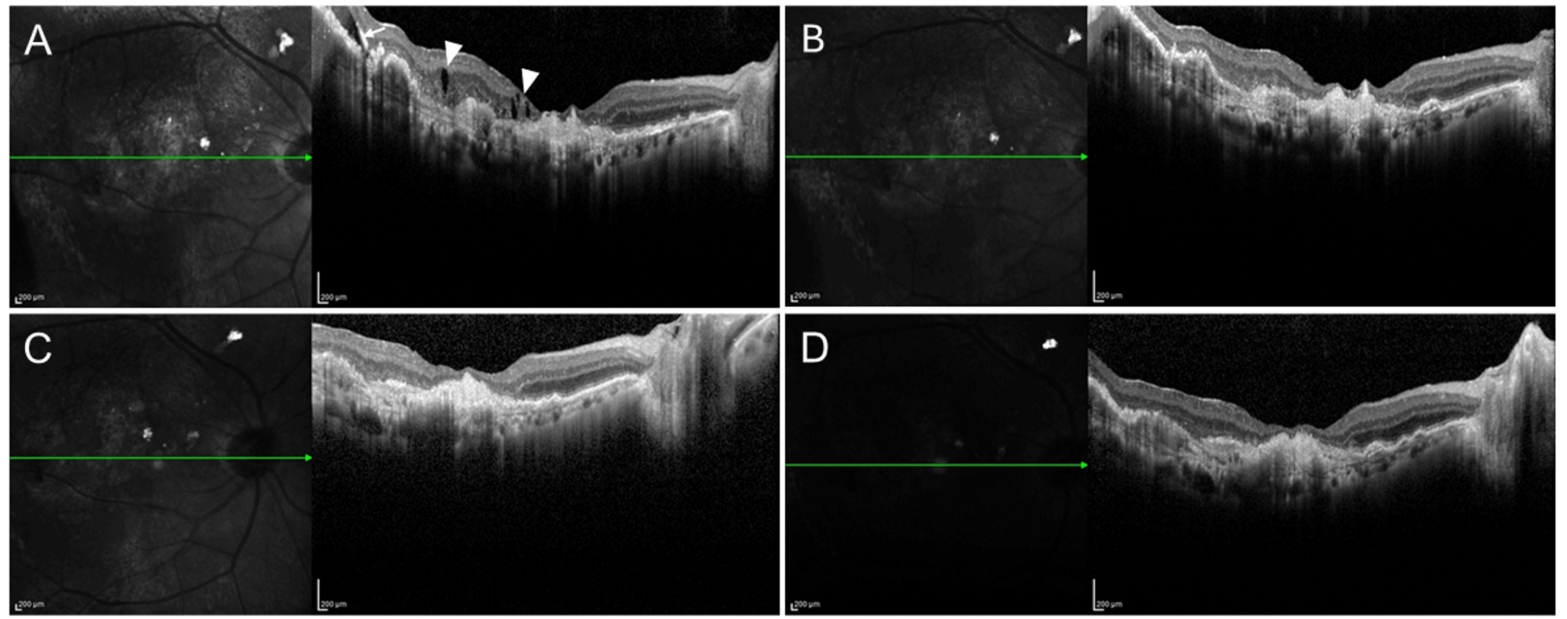
OCT images of a vitrectomized eye with nAMD (A) Five weeks after the administration of aflibercept 2 mg, IRF (arrowheads) and SRF (arrow) were observed; VA was 20/32. (B) Five weeks after switching to aflibercept 8 mg, no IRF or SRF was observed; the VA was 20/25. (C) Five weeks after the second aflibercept 8 mg injection: a dry macula was achieved; the VA was 20/25. (D) Eight weeks after the third aflibercept 8 mg injection: a dry macula was maintained; the VA was 20/25. IRF, intraretinal fluid; SRF, subretinal fluid; OCT, optical coherence tomography; nAMD, neovascular age-related macular degeneration; VA, visual acuity

Despite continuous administration of aflibercept 2 mg every five weeks, persistent exudation remained, and best-corrected visual acuity (BCVA) was 20/32 (Figure 1A). Switching to aflibercept 8 mg led to complete resolution of exudation at the same five-week interval, and the BCVA improved to 20/25 (Figure 1B-1C). A dry macula and a BCVA of 20/25 were maintained after extending the interval to eight weeks (Figure 1D).

Case 2

**Figure 2:**
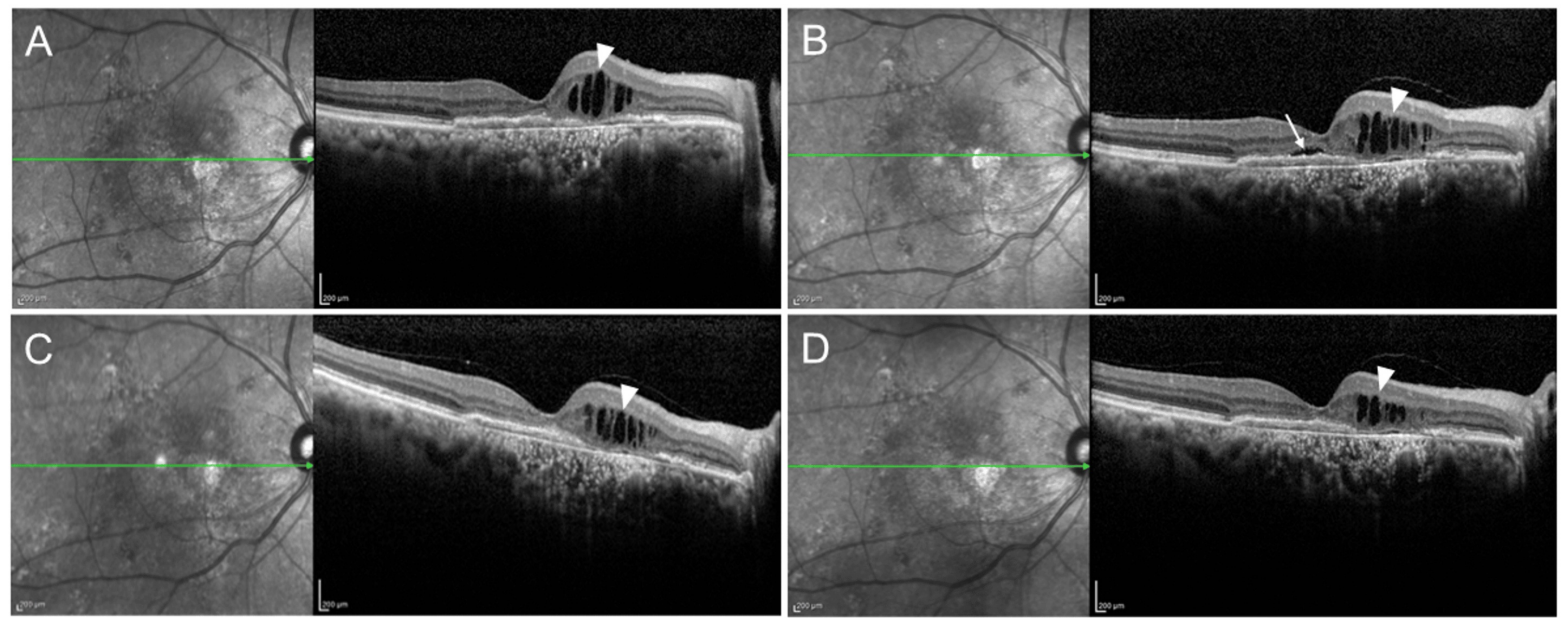
OCT images of a vitrectomized eye with nAMD (A) Six weeks after aflibercept 2 mg administration: IRF (arrowhead) was observed, SRF was absent; VA was 20/40. (B) Eight weeks after aflibercept 2 mg administration: SRF (arrow) appeared; VA was 20/32. (C) Eight weeks after switching to aflibercept 8 mg: SRF resolved; VA was 20/32. (D) Eight weeks after the second aflibercept 8 mg injection: absence of SRF was maintained; VA was 20/40. IRF, intraretinal fluid; SRF, subretinal fluid; OCT, optical coherence tomography; nAMD, neovascular age-related macular degeneration; VA, visual acuity

A 77-year-old man with a type 1 MNV underwent PPV with intrascleral IOL fixation for zonular rupture. Over 7.5 years of nAMD treatment, he received 45 aflibercept 2 mg injections. At six-week intervals, no subretinal fluid (SRF) was observed, and BCVA was 20/40 (Figure 2A); however, SRF recurred and BCVA improved to 20/32 when the interval was extended to eight weeks (Figure 2B). After switching to aflibercept 8 mg, the SRF resolved at the same eight-week interval, and BCVA was maintained at 20/32 (Figure 2C). The absence of SRF was preserved, and BCVA was stable at 20/40 after the second injection (Figure 2D).

Case 3

**Figure 3:**
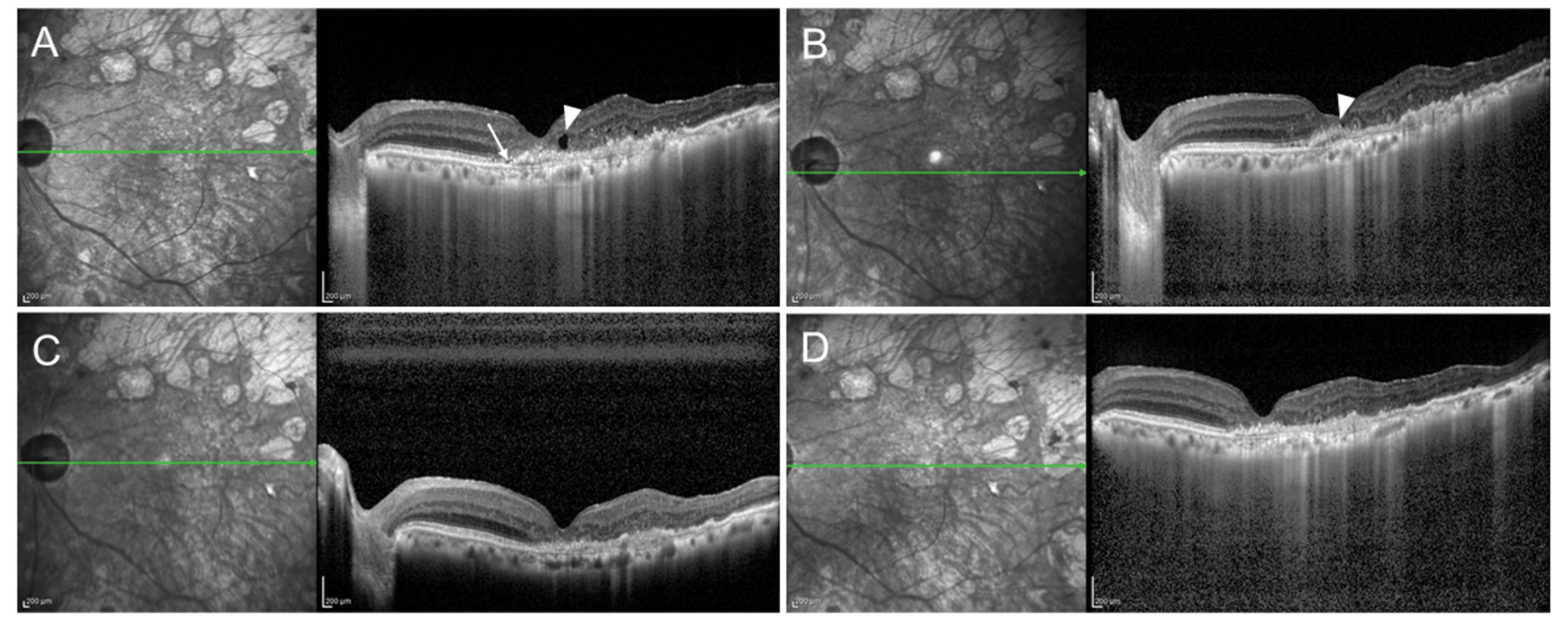
OCT images of a vitrectomized eye with nAMD (A) Eight weeks after aflibercept 2 mg administration: IRF (arrowhead) and SRF (arrow) were observed; VA was 20/32. (B) Six weeks after aflibercept 2 mg administration: IRF decreased compared to the 8-week interval; SRF was absent; VA was 20/32. (C) Eight weeks after switching to aflibercept 8 mg: IRF resolved; VA was 20/32. (D) Eight weeks after the second aflibercept 8 mg injection: a dry macula was maintained; VA was 20/40. IRF, intraretinal fluid; SRF, subretinal fluid; OCT, optical coherence tomography; nAMD, neovascular age-related macular degeneration; VA, visual acuity

An 83-year-old woman underwent PPV and cataract surgery for an epiretinal membrane secondary to a branch retinal vein occlusion. Ten years later, she was diagnosed with polypoidal choroidal vasculopathy and received 10 injections of aflibercept 2 mg over 1.5 years. At eight-week intervals, both SRF and intraretinal fluid (IRF) were present, and BCVA was 20/32 (Figure 3A). Shortening the interval to six weeks reduced IRF, eliminated SRF, and maintained the BCVA at 20/32 (Figure 3B). Switching to aflibercept 8 mg resulted in complete IRF resolution at eight-week intervals, with BCVA of 20/32 (Figure 3C). A dry macula and a BCVA of 20/40 were maintained after the second injection (Figure 3D).

## Discussion

We report three vitrectomized eyes with nAMD in which switching from aflibercept 2 mg to aflibercept 8 mg resulted in anatomical improvement and allowed interval extension. These findings suggest that aflibercept 8 mg can provide enhanced durability even in eyes in which drug clearance may be accelerated.

However, the pharmacokinetic effects of vitrectomy on intravitreal anti-VEGF agents remain unclear. Although some animal studies have reported unchanged clearance [14,15], others have reported significantly faster clearance after PPV [16,17]. These discrepancies may stem from differences in PVD completeness, residual vitreous gel, surgical techniques, or concomitant lensectomy [9]. All our patients underwent complete PVD induction and lens extraction, potentially increasing the clearance risk; however, aflibercept 8 mg achieved sufficient VEGF suppression.

The PULSAR trial demonstrated the non-inferiority of aflibercept 8 mg administered every 12 or 16 weeks compared with 2 mg every eight weeks in treatment-naïve nAMD, with over 80% of patients maintaining ≥12-week intervals [5]. Real-world data have also indicated significant efficacy and durability in reducing exudative changes and extending treatment intervals with 8 mg [18,19]. However, vitrectomized eyes were excluded, and our cases suggest that, despite possible accelerated clearance, a higher dose can maintain efficacy in this subgroup.

No ocular inflammation was observed, alleviating concerns about higher molar concentrations and increased inflammatory risk. In addition, long-term follow-up is warranted to monitor the development and progression of macular atrophy. Our study was limited by its retrospective, single-center design, small sample size, and short follow-up period. Larger prospective studies are required to validate these findings.

## Conclusions

In our small series, switching from aflibercept 2 mg to aflibercept 8 mg in vitrectomized eyes with nAMD resulted in both anatomical improvement and treatment interval extension without safety concerns. Aflibercept 8 mg may be a viable strategy for patients requiring frequent injections after vitrectomy to reduce the treatment burden. Larger prospective studies are required to confirm these findings.
